# Neuroimmunological processes in Parkinson's disease and their relation to α-synuclein: microglia as the referee between neuronal processes and peripheral immunity

**DOI:** 10.1042/AN20120066

**Published:** 2013-04-30

**Authors:** Vanesa Sanchez-Guajardo, Christopher J. Barnum, Malú G. Tansey, Marina Romero-Ramos

**Affiliations:** *CNS Disease Modeling Group, Department of Biomedicine, Ole Worms Allé 3, Aarhus University, DK-8000 Aarhus C, Denmark; †Department of Physiology, Emory University, School of Medicine, Atlanta, GA 30233, U.S.A.

**Keywords:** lymphocytes, M1/M2 phenotype, microglia, neuroinflammation, Parkinson’s disease, α-synuclein, 6-OHDA, 6-hydroxydopamine, AD, Alzheimer’s disease, APC, antigen-presenting cell, α-syn, α-synuclein, BBB, brain–blood barrier, BCG, Bacille Calmette–Guérin, BM, bone marrow, CFA, complete Freund’s adjuvant, CM, conditioned media, CNS, central nervous system, COX, cyclooxygenase, CR, complement receptor, CSF, cerebrospinal fluid, DA, dopamine, EAE, experimental autoimmune encephalomyelitis, GA, galatiramer acetate, GDNF, glial-derived neurotrophic factor, GFP, green fluorescent protein, HLA-DR, human leucocyte antigen type DR, IFNγ, interferon γ, IgG, immunoglobulin G, IL, interleukin, iNOS, inducible nitric oxide synthase, LAMP, lysosome-associated membrane protein, LB, Lewy body, LPS, lipopolysaccharide, MHC, major histocompatibility complex, MPTP, 1-methyl-4-phenyl-1,2,3,6-tetrahydropyridine, NFκB, nuclear factor κB, NK, natural killer, NO, nitric oxide, PD, Parkinson’s disease, PET, positron-emission tomography, PrP, prion protein, rAAV, recombinant adeno-associated virus, RNS, reactive nitrogen species, ROS, reactive oxygen species, SN, substantia nigra, SNP, single nucleotide polymorphism, TCR, T-cell receptor, TGFβ, tumour growth factor β, TH, tyrosine hydroxylase, Th1, T helper 1, TLR, Toll-like receptor, TNF, tumour necrosis factor, T_reg_, regulatory T-cell, VIP, vasoactive intestinal peptide, WT, wild-type

## Abstract

The role of neuroinflammation and the adaptive immune system in PD (Parkinson's disease) has been the subject of intense investigation in recent years, both in animal models of parkinsonism and in post-mortem PD brains. However, how these processes relate to and modulate α-syn (α-synuclein) pathology and microglia activation is still poorly understood. Specifically, how the peripheral immune system interacts, regulates and/or is induced by neuroinflammatory processes taking place during PD is still undetermined. We present herein a comprehensive review of the features and impact that neuroinflamation has on neurodegeneration in different animal models of nigral cell death, how this neuroinflammation relates to microglia activation and the way microglia respond to α-syn *in vivo*. We also discuss a possible role for the peripheral immune system in animal models of parkinsonism, how these findings relate to the state of microglia activation observed in these animal models and how these findings compare with what has been observed in humans with PD. Together, the available data points to the need for development of dual therapeutic strategies that modulate microglia activation to change not only the way microglia interact with the peripheral immune system, but also to modulate the manner in which microglia respond to encounters with α-syn. Lastly, we discuss the immune-modulatory strategies currently under investigation in animal models of parkinsonism and the degree to which one might expect their outcomes to translate faithfully to a clinical setting.

## OVERVIEW: NEUROINFLAMMATION AND MICROGLIA ACTIVATION IN PD (PARKINSON’S DISEASE)

The initial observation that activated microglia were detectable in brains from PD patients at autopsy (McGeer et al., 1988) came 25 years ago. Since then, numerous studies in both humans and animal models of parkinsonism have implicated inflammatory processes in the development and progression of nigral dopaminergic neuron death [for a detailed review (Tansey and Goldberg, 2010)]. Most noteworthy is the recent proposal that neuroinflammation is likely to play a key role in propagation of misfolded α-syn (α-synuclein) in a ‘prion-like’ fashion in PD (Lema Tome et al., 2012). In this review, we propose and discuss the idea that, over the course of PD, the initially neuroprotective microglia becomes toxic to DA (dopamine) neurons as a result of overproduction of ROS/RNS (reactive oxygen species and reactive nitrogen species) and cytokines. We also explore the notion that, in parallel to these processes, microglia engage peripheral immune cells to act on the brain, resulting in a dynamic cross-regulation of their respective phenotypes.

Enhanced microglia activation in the PD brain is likely to be occurring prior to the death of nigral DA neurons and in parallel with neuronal dysfunction and loss of DA terminals. In support of this idea, *in vivo* imaging studies of microglial activation with the peripheral benzodiazepine receptor binding ligand [11C]-(R) PK11195 using PET (positron-emission tomography) showed that, irrespective of the number of years with the disease, patients with idiopathic PD have markedly elevated neuroinflammation in the pons, basal ganglia, striatum and frontal and temporal cortical regions compared with age-matched healthy controls (Gerhard et al., 2006). Therefore microglia that become activated early in the disease process (by triggers discussed in other sections of this review) may remain primed, leaving them poised to respond robustly and/or aberrantly to subsequent stimuli (including dying neurons) thereby enhancing inflammation-induced oxidative stress in vulnerable brain regions. Indeed, phagocytic activity of microglia during debris removal is associated with respiratory bursts and would be expected to further enhance oxidative stress for the remaining population of DA neurons, while homoeostatic ‘nibbling’ of synapses by microglia are known to regulate neuronal transmission and maintain neuronal health. Importantly, microglia-derived factors and/or release of chemoattractants by the dying DA neurons (Aloisi, 2001; Kim and de Vellis, 2005; Sriram et al., 2006) are likely to play a role in recruitment of peripheral immune cells and influence PD progression. The protective compared with detrimental role of the peripheral immune system in PD pathophysiology is an area of investigation that we will discuss in this review.

## INFLAMMATORY SIGNS IN PD PATIENTS

Several features in both brain and peripheral blood support a role for the immune system in PD. Within the brain, PET imaging of PD patients has revealed that microglia are active not only within the SN (substantia nigra) but also in all brain areas implicated in PD (Ouchi et al., 2005; Gerhard et al., 2006). This is supported by the post-mortem immunohistological analysis of PD brains that show morphological changes in microglia and up-regulation of specific proteins such as HLA-DR^+^ (human leucocyte antigen type DR) that relate to differences in function/activation (McGeer et al., 1988; Imamura et al., 2003; Croisier et al., 2005; Orr et al., 2005). This last finding suggests the possibility that microglia activation could be a surrogate marker for early PD pathology as up-regulation of HLA-DR expression appears to be an early pathological event in the disease process. Another activation marker up-regulated in the brains of PD patients and widely used in animal models of PD is the phagocytic receptor CD68, also known as macrosialin, which upon microglia activation is often found in cytoplasmic vesicles (Banati et al., 1998; Croisier et al., 2005). Other proteins related to microglia induction of neuroinflammation are also increased within the brains of PD patients, such as COX (cyclooxygenase) and iNOS (inducible nitric oxide synthase) (Hunot et al., 1996; Knott et al., 2000).

The adaptive immune system has also been implicated in PD pathophysiology, as CD4/CD8 T-cells infiltrate the SN of PD patients (McGeer et al., 1987, 1988; Farkas et al., 2000; Brochard et al., 2009) and may contribute to vascular changes during the disease (Faucheux et al., 1999; Farkas et al., 2000). Moreover, it appears that the peripheral T-cell pool is also altered during PD (Hisanaga et al., 2001; Baba et al., 2005). In particular, the CD4+ population has been found to decrease (Bas et al., 2001; Calopa et al., 2010). The reasons for this decline are unknown but likely result from increased DNA oxidative damage (Migliore et al., 2002; Cornetta et al., 2009) and induction of apoptosis (Blandini et al., 2003; Calopa et al., 2010). Of particular interest to our group is the fact that CD4+ γδT-cells, which are mainly activated locally and not in secondary lymphoid organs, are increased in the periphery as well as in the CSF (cerebrospinal fluid) of PD patients where they display an activated phenotype (Fiszer, 1989).

A role for humoral immunity has also been proposed in PD progression. LB (Lewy body) in PD brains shows strong immunolabelling for IgG (immunoglobulin G) and about one-third of SN DA neurons show surface immunoreactivity for IgG. Interestingly, the proportion of IgG immunopositive neurons positively correlated with the number of HLA-DR+ microglia and negatively correlated with the number of remaining DA neurons in SN, suggesting that surface coating of DA neurons with IgG may target them for degradation early in the disease process (Orr et al., 2005). In addition, antibodies against α-syn have been found in serum and CSF of patients with certain forms of familial PD (Papachroni et al., 2007). A recent study showed that sera from patients with sporadic PD contained disease-specific auto-antibodies (Han et al., 2012) and antibodies from CSF of PD patients were cross-reactive with rat neurons (McRae Degueurce et al., 1986) as well as with proteins modified by DA oxidation (Rowe et al., 1998).

For two decades, PD researchers have known about the presence of elevated levels of cytokines [including TNF (tumour necrosis factor), IL (interleukin)-1β, IL-2, IL-4 and IL-6] in post-mortem SN of PD patients (Mogi et al., 1994a, 1994b; Hunot and Hirsch, 2003). The simplest interpretation of these observations was that the neuroinflammatory response was an end-stage result of microglia activation following neuronal death. However, they also suggested the possibility that the local environment created by cytokine signalling impacted survival of nigral DA neurons and could affect the course of PD. Specifically, levels of TNF in the healthy adult brain are generally very low and produced primarily by neurons (Breder et al., 1993); however, in the area of maximal destruction where the vulnerable melanized DA-producing neurons reside in the ventral midbrain, the levels of TNF, IL-1β and IFNγ (interferon γ) are significantly increased in PD patients compared with normal controls (Hirsch et al., 1998). In addition to elevated CNS (central nervous system) levels, elevated cytokine levels in the peripheral circulation of PD patients have also been reported (Koziorowski et al., 2012) and may underlie some non-motor symptoms of PD. Specifically, the levels of TNF in the serum of PD patients were found to be more elevated in patients with more severe symptoms of depression and fatigue (Lindqvist et al., 2012) as well as impaired cognition and sleep disturbances (Menza et al., 2010). Although the CSF of PD patients has been reported to contain high concentrations of IL-1β (Blum-Degen et al., 1995; Mogi et al., 1996), this finding is not specific as brains of patients with AD (Alzheimer's disease) and LB dementia also display IL-1β-expressing microglia within the vicinity of neurons that were highly immunoreactive for βAPP (β-amyloid precursor protein) and contained both LBs and neurofibrillary tangles (Grigoryan et al., 2000). In fact, these observations raise the possibility that the clinical and neuropathological overlap between AD and PD could be mediated by IL-1β (Mrak and Griffin, 2007). Although the number of studies implicating pro-inflammatory cytokines in PD progression is numerous, increased levels of other cytokines with anti-inflammatory or repair functions such as IL-10 have also been reported in patients with PD (Mogi et al., 1996; Nagatsu et al., 2000; Brodacki et al., 2008). Given the already successful use of anti-TNF biologics in the treatment of rheumatoid arthritis, inflammatory bowel disease and psoriasis, it is not unreasonable to think that CNS delivery of such agents may afford therapeutic benefit to patients with PD or other neurological disorders characterized by chronic neuroinflammation (Clark et al., 2010).

The genes for various cytokines, chemokines and acute phase proteins have been surveyed in attempts to find associations between specific SNPs (single nucleotide polymorphisms) and incidence of early or late-onset PD (Nishimura et al., 2001; Ross et al., 2004; Wahner et al., 2007; Wu et al., 2007; Bialecka et al., 2008; Infante et al., 2008; Pascale et al., 2011). With regards to TNF, a significant association between certain (but not all) SNPs in the TNF promoter and PD has been reported recently (Chu et al., 2012). Additional studies will be necessary to validate these findings in order to assess the overall genetic effect of TNF gene polymorphisms in human populations. Although the endogenous levels of IFNγ in healthy human brain are known to be virtually undetectable (Frugier et al., 2010), allelic differences between early- and late-onset PD patients were reported for the *IFNγ* gene, a provocative finding that may influence infiltration of T-cells during progression of the disease since T-cells are the major producers of IFNγ. In contrast, certain studies find that specific IL-1β promoter polymorphisms lower the risk of PD (Nishimura et al., 2001, 2005). However, a recent meta-analysis did not find any such association (Chu et al., 2012). Finally, the IL-10 promoter polymorphism-819 has been associated with higher risk for early onset PD but not for sporadic PD (Li et al., 2012) and the G1082A SNP has been associated with age of disease onset (Hakansson et al., 2005), whereas other studies showed no correlation between polymorphisms-1082 or -592 with any type of PD (Bialecka et al., 2008; Pascale et al., 2011; Chu et al., 2012).

All in all, data from patients support a complex role for the immune system and inflammatory factors in PD; in particular microglia, which are probably actively involved in various disease processes rather than being mere scavengers of cellular debris. Moreover, the complexity of the human disease and the interactions of inflammatory pathways will probably mean that a successful intervention to protect the nigrostriatal pathway from death-inducing inflammatory insults and/or treat non-motor symptoms arising from chronic neuroinflammation will very likely require a multi-target immunomodulatory approach. In addition, these immunomodulatory interventions are likely to be more efficacious in the earliest stages of PD to promote an M2 microglia phenotype over an M1 phenotype and increase the levels of protective cytokines while minimizing the levels of cytotoxic pro-inflammatory cytokines.

## ROLE OF MICROGLIA ACTIVATION IN RODENT MODELS OF NIGRAL DOPAMINERGIC CELL DEATH

### MPTP (1-methyl-4-phenyl-1,2,3,6tetrahydropyridine)

Three decades after the initial observation that faulty chemistry during an attempt to make synthetic opiates resulted in formation of MPTP, the MPTP model remains one of the oldest and most widely used neurotoxins to induce parkinsonism (in particular nigral cell death) in mice and primates. MPTP is a pro-toxin that gets converted into MPP+ (N-methyl-4 phenylpyridinium) by monoamine oxidase-B enzyme within astrocytes (Ransom et al., 1987). It is subsequently taken up by DA neurons and interacts with the mitochondrial respiratory chain and damages complex-1, leading to cell death (Williams and Ramsden, 2005). Concomitantly, inflammatory cytokines such as TNF and ROS/RNS are increased (Smeyne and Jackson-Lewis, 2005; Miller et al., 2009) raising the possibility that inflammation may contribute to MPTP-induced nigral cell death. Post-mortem examination of human subjects exposed to MPTP, revealed the presence of activated microglia several years after drug exposure (Langston et al., 1999) and similar findings have been reported in non-human primates (McGeer et al., 2003; Barcia et al., 2004), suggesting that even a single exposure to MPTP can induce a persistent inflammation. Interestingly, both in MPTP-treated non-human primates and in mice, the outcome of MPTP intoxication is strain-dependent. Specifically, the motor deficits associated with gliosis and loss of striatal TH (tyrosine hydroxylase) occur in MPTP-treated C57/Bl6 mice but not in Balb/c (Yasuda et al., 2008). One possible reason for this could be that the peripheral immune system in the C57/Bl6 strain is prone to a Th1 (T helper 1) phenotype (pro-inflammatory, IFNγ producing), whereas Balb/c mice are prone to mount a Th2 (anti-inflammatory) immune response. These interesting differences suggest a modulatory role for the peripheral immune response in MPTP-induced degeneration and raise the distinct possibility that neuroinflammatory processes may compromise and/or hasten nigral degeneration induced by oxidative neurotoxins.

A recent study aimed at identifying the extent to which microglia activation contributes to the effects of MPTP in monkeys indicated that microglial activation is triggered early mainly by the toxic effects of MPTP regardless of the dose (subacute or chronic) used and the extent of cell death induced (Vazquez-Claverie et al., 2009). However, as in humans, this microgliosis persisted 35 months after the last MPTP intoxication and in all cases was associated with up-regulated HLA-DR expression in microglia (Vazquez-Claverie et al., 2009). It should be noted that despite the similarities in microglia HLA-DR expression triggered by MPTP and that present in the PD brain, many other phenotypic differences are likely to exist between the two microglioses, such as their profiles of inflammatory factor production. In fact, conflicting findings on the neuroprotective effects of anti-inflammatory agents suggest that the role of inflammation in MPTP models is quite complex (see the section on immunomodulation as a therapy for PD). Nevertheless, the strongest support for inflammatory involvement in MPTP-induced DA cell death was demonstrated using iNOS-null mice, which were considerably more protected from MPTP-induced DA cell death than WT (wild-type) mice (Liberatore et al., 1999). Other studies have also shown that inhibition of iNOS activity attenuated nigral degeneration following MPTP administration (Dehmer et al., 2000). While many of the inflammatory targets that participate in MPTP-induced nigral degeneration have yet to be identified, the general consensus is that inflammation is likely to play an important modulatory role.

### 6-OHDA (6-hydroxydopamine)

The unilateral 6-OHDA model of hemiparkinsonism has been the gold standard rat model of nigral cell death since it was first used more than 40 years ago (Ungerstedt and Arbuthnott, 1970). A neurotoxic analogue of DA, 6-OHDA selectively kills DA and noradrenaline (norepinephrine) neurons when it is injected in the striatum and taken up from the extracellular space by their respective transporters DAT and NET (Luthman et al., 1989). The primary mechanism by which 6-OHDA induces cell death is through oxidative stress, although inhibition of mitochondrial respiration has also been noted and when administered *in vivo*. There is an abundance of evidence that 6-OHDA is toxic to DA neurons in part through inflammatory mechanisms (see Schober, 2004 for review). PET imaging with PK11195, a marker of activated microglia, revealed increased microglial activity within the SN following 6-OHDA intrastriatal injection (Cicchetti et al., 2002). This observation is supported by other studies in which microglia and inflammatory mediators play an important role in 6-OHDA-induced degeneration (Mogi and Nagatsu, 1999; Wilms et al., 2003a, 2003b; Nagatsu and Sawada, 2005; McCoy et al., 2006). Moreover, the inflammatory profile observed in 6-OHDA-lesioned animals may also depend on the site of injection. For example, when 6-OHDA is injected into the striatum, microglial activation appears to be more robust within the striatum than in the SN at 7 and 28 days after the lesion (Armentero et al., 2006). In contrast, others (Na et al., 2010) reported that intrastriatal 6-OHDA resulted in an increase in inflammatory-related gene expression within both the striatum and the SN 7 days post-lesion, an effect that persisted within the SN for 14 days. Taken together, data from multiple studies suggest that inflammatory processes play a secondary but important role in 6-OHDA-induced nigral degeneration.

### LPS (lipopolysaccharide)

LPS-induced inflammatory signalling has been shown to compromise survival of DA neurons and has been described as an inflammatory model of parkinsonism in rodents (Castano et al., 1998; Ferrari et al., 2006; Barnum and Tansey, 2010). LPS is a gram-negative bacterial endotoxin that activates inflammatory responses through the TLR (Toll-like receptor) 4, which is highly expressed in microglia. Over the years, researchers have administered LPS *in vivo* in a variety of ways to investigate its effects on the nigrostriatal pathway. LPS has been injected within the CNS (intraventricular, intrastriatal and intranigral) and systemically (intraperitoneal), acutely and/or chronically and even prenatally (described in more detail below). In each instance, LPS-induced inflammatory signals resulted in selective toxicity for DA neurons despite the fact that TLR4 receptor expression is undetectable in isolated DA neurons from ventral mesencephalon. Several studies have reproducibly demonstrated that a single intranigral injection of LPS can activate microglia and selectively reduce the number of DA neurons in the ventral midbrain (Castano et al., 1998). Interestingly, in a follow-up study authors demonstrated that LPS-induced nigral cell death could be attenuated by peripheral administration of dexamethasone, a synthetic glucocorticoid receptor antagonist with potent anti-inflammatory properties but poor brain penetration (Castano et al., 2002) and by central administration of soluble TNF inhibitors (Mogi and Nagatsu, 1999; Wilms et al., 2003a, 2003b; Nagatsu and Sawada, 2005; McCoy et al., 2006). On the one hand, these findings suggest that the BBB (blood–brain barrier) may be compromised as a result of central LPS administration, but more importantly they raise the interesting possibility that modulation of peripheral inflammation can have effects in the CNS. Moreover, it is important to recognize that not all the inflammatory responses triggered by central administration of LPS compromise nigral DA neuron survival. Specifically, acute administration of LPS was recently reported to increase IL-1β expression and trigger production of GNDF (glial-derived neurotrophic factor) from astrocytes (Iravani et al., 2012). Similarly, chronic low-dose intranigral administration of LPS via osmotic pumps in rats was reported to induce neuroinflammation in the CNS and resulted in delayed and progressive loss of nigral DA neurons *in vitro* and *in vivo* (Gao et al., 2002). The DA cell loss appeared to be permanent (no recovery after 12 months) and was specific to DA cells while sparing GABA (γ-aminobutyric acid) ergic and serotonergic cells (Herrera et al., 2000). Moreover, nigral cell death has also been elicited by a single injection of LPS peripherally (Qin et al., 2007) or pre-natally (Carvey et al., 2003). Finally, chronic low-dose intraperitoneal LPS injections have been shown to act in concert with parkin deficiency and induce nigral DA neuron loss (Frank-Cannon et al., 2008), suggesting that a non-specific immunogenic stimulus such as LPS can act in concert with genetic susceptibility genes and selectively compromise survival of DA neurons. In summary, multiple studies indicate that chronic or acute LPS administration in rodents can hasten selective and progressive loss of nigral DA neurons. Since these paradigms reproducibly elicit delayed and selective death of 30–70% of nigral DA neurons, inflammatory models of nigral cell death offer unique opportunities to study the molecular mechanisms that may contribute to progressive loss of DA neurons akin to that occurring in patients with PD.

### Rotenone

The pesticide rotenone has been directly linked to idiopathic PD (Tanner et al., 2011) and chronic administration in rats was reported to induce microglia activation (Sherer et al., 2003) and selective degeneration of DA neurons in SN (Betarbet et al., 2000). Although the original delivery method (osmotic minipump and indwelling cannulae into midbrain) was fraught with large variability and lack of reproducible effects *in vivo*, the modified model of intraperitoneal injection of rotenone at 3 mg kg^−1^ day^−1^ in a neutral inorganic oil such as Miglyol (Cannon et al., 2009) has been reported to yield very consistent results in rats (Martinez and Greenamyre, 2012). More recently, a chronic rotenone mouse model was reported but the neuroinflammatory responses in that model have not yet been well characterized (Inden et al., 2011). Although it is clear that application of rotenone *in vivo* triggers microglia activation and induces loss of nigral DA neurons, rotenone does not cause direct activation of microglia *in vitro* (Gao et al., 2002, 2003; Shaikh and Nicholson, 2009; Klintworth et al., 2009). Based on these findings, some have suggested its toxicity is in part related to its ability to disturb the CD200R-CD200L microglia-DA neuron cross-talk in the midbrain (Wang et al., 2011).

## ROLE OF MICROGLIA ACTIVATION IN α-SYN-INDUCED DEGENERATION

### Microgliosis and α-syn pathology in brain

In 1997, Polymeropoulos et al. first described a family with an inherited form of PD carrying a mutation in the SNCA gene (Polymeropoulos et al., 1997). This gene encodes the protein α-syn, one of three members of a gene family that includes a β- and γ-syn. Shortly thereafter, α-syn was shown to be the main component of LB and LN (Lewy neurites) in PD patients (Spillantini et al., 1997). Since this original finding, two more SNCA mutations have been genetically linked to familial PD (Kruger et al., 1998; Zarranz et al., 2004) as well as gene triplication (Ross et al., 2008), indicating that an excessive amount of this normal protein can also cause PD. This discovery led to the generation of multiple transgenic mouse lines based on overexpression of the WT or mutated protein under various heterologous promoters (for a review see Chesselet and Richter, 2011). These transgenic mouse lines have been very useful in our quest to understand the physiological role of α-syn, disease complexity, and early events that may contribute to neuronal degeneration, despite the fact that none of the lines display robust DA neurodegeneration. In addition, overexpression models based on adult transgenesis of α-syn by means of viral vectors have achieved significant cell death in SN and therefore a PD-like motor and neuropathological profile (Ulusoy et al., 2010).

Animal models have confirmed the dose-dependent toxicity of α-syn and its ability to induced neurodegeneration not only in dopaminergic neurons but also in other neuronal populations. Braak et al. (2003) observed the multifactorial features and complexity of disease progression based on α-syn pathology in PD patients’ brains, and suggested that pathology may start in the olfactory nucleus and several dorsal motor nuclei and in time progress as α-syn pathology spreads upwards into midbrain and cortex. In this regard, the presence of activated microglia in the PD brain has been regarded as a sensitive index of neuropathological changes that are present not only in SN but also in other brain regions such hippocampus and cortex (Imamura et al., 2003). It should be noted that microglia form a heterogeneous population as revealed by the differences in the expression of surface markers between microglia from different CNS regions (de Haas et al., 2008). This diversity is probably influenced by the result of local cues such as neuronal activity (Neumann, 2001) and the activity of neurotransmitters such as DA and noradrenaline, given that microglia express receptors for both (Farber et al., 2005). The integrity of the BBB will also very likely impact the role of microglia as microglia-derived factors such as chemokines are likely to increase trafficking of peripheral immune cells to the CNS; and in PD there are some indications of BBB dysfunction/changes as the disease progresses (Kortekaas et al., 2005; Desai et al., 2007; Pisani et al., 2012) although this seems to be a late event (Haussermann et al., 2001). In addition, it is likely that α-syn-associated pathology also modulates the microglia response as α-syn deposition correlates with the presence of HLA-DR [human homologue of MHC II (major histocompatibility complex II)] expressing microglia (Croisier et al., 2005). The neuron–microglia interaction can result from changes in neurons that are subsequently sensed by microglia or vice versa, creating a feed-forward mechanism that contributes to the maintenance of neuroinflammation and progression of disease. Therefore neurons expressing α-syn can activate microglia (see below). In turn, microglia enhance the local inflammatory environment surrounding α-syn-expressing neurons and may lead to abnormal handling of α-syn in neurons. Presence of abnormal α-syn expression in cells surrounding neuroinflammatory lesions within the brains of patients with multiple sclerosis supports the idea that the neuron–microglia interaction that exacerbates the disease process can be initiated either by the microglia or the neuron (Lu et al., 2009). Although the neuronal and microglial changes may be two independent processes occurring in parallel, there is significant evidence of cross-talk between these two populations under homoeostatic conditions that could become disrupted during PD. Proteins known to mediate neuron–glia cross-talk via direct protein–protein interactions include CD200–CD200L, CD45–CD22 and fractalkine-CX3CR1 as well as many other different microglia- and neuron-derived factors that can shape the response or function of both cell populations (for a review see Bessis et al., 2007; Biber et al., 2007, 2008).

In addition, several markers previously thought to be only associated with neurons or microglia have now been shown to be relevant in both types of cells. For example, increased expression of NFκB (nuclear factor κB) in the SN of PD patients is found in CD11b+ microglia and also in affected neurons (Ghosh et al., 2007). Similarly, caspase activation is involved not only in neuronal cell death but also in microglia activation (Burguillos et al., 2011), strengthening the idea that responses from both populations are involved in disease progression. In that sense, the environment created by the immune system will very likely affect α-syn levels. As noted in an earlier section, there are well-documented changes in cytokines in PD patients, both in the periphery and in the CNS (for an in-depth review see Tansey and Wyss-Coray, 2008). In parallel, it has been shown that the level of α-syn in human microglia is regulated by cytokine exposure (Bick et al., 2008). Moreover, exposure of microglia to CSF from PD patients resulted in an increase in intracellular α-syn (Schiess et al., 2010) although it was not determined whether this was due to increased α-syn uptake or up-regulation of α-syn expression by microglia. It is our view that the microenvironment present in a PD brain will favour accumulation of α-syn in microglia that in turn causes them to respond by becoming activated, as suggested by multiples studies reviewed below.

### Microgliosis in α-syn animal models of nigral cell death

Regarding the various α-syn transgenic mouse lines, few groups have done in-depth analyses of the neuroinflammatory profiles, but many report microgliosis as a common pathological finding (for a review see Magen and Chesselet, 2010). Microgliosis, defined as an increase in microglia number, change in activation state based on morphological characteristics, and the presence of inflammatory markers, has been observed prior to the onset of cell death and coinciding with cellular dysfunction (often marked by down-regulation of TH without frank neuronal loss) in different animal models (Table 1). These observations support a role for innate immune responses early in the disease process and argue against a role for microglia as mere scavengers of neuronal debris. A detailed study investigating early and late microgliosis was reported recently in the Thy-1 WT α-syn line. In this study, microgliosis was first observed in the striatum and progressed to the SN and preceded motor deficits, highlighting the susceptibility of DA neurons (Watson et al., 2012). Overexpression of a C-terminal truncated α-syn (shown to aggregate faster *in vitro*) induced activation of microglia in the absence of TH+ cell death but in regions where neurodegenerative changes were evident (Tofaris et al., 2006). Microgliosis was found in areas such as cortex and hippocampus of A30P overexpressing mice under the PrP (prion protein) promoter along with the presence of truncated and oligomeric α-syn in such areas (Gomez-Isla et al., 2003). Also, α-syn pathological accumulation has been associated with microgliosis in the E46K α-syn transgenic under the PrP (Emmer et al., 2011). Finally, the A53T α-syn transgenic mouse line under the PrP promoter and the A30P+A53T α-syn line under the TH promoter show changes in microglia cell numbers and altered expression patterns in multiple genes related to the inflammatory responses (Lee et al., 2002; Miller et al., 2007).

**Table 1:** *In vivo* studies on effects of α-synuclein on microglia ICAM, intercellular adhesion molecule; m, month; rAAV, recombinant adeno-associated virus; α-Syn, α-Synuclein; SN, substantia nigra; str, striatum; TH, tyrosine hydroxylase; w, weeks; WB, Western blots; *DM (double mutant) not occurring in humans; in italics, markers included in the studies that did not show any change. Unless otherwise noted, α-syn used was human.

Reference	α-Syn	*In vivo* approach	Animals	Observations	Notes
Wilms et al., 2009	Oligomeric WT 0.5 ng	Direct intra-SN injection	Wistar rat	↑Iba-1+ and 22% TH+ cell loss in SN after 1w	Protected by MAPK inhibitor semapimod
Yu et al., 2010	Nitrated TAT α-syn 0.84 μg	Direct intra-SN injection	Sprague–Dawley rat	↑Iba-1+ and GFAP+ cells in SN after 5w	TH cell death observed after 5w 34.5% and 11w 48.7%. If not nitrated, no cell death
Couch et al., 2011	WT 3 μg	Direct intra-SN injection	Mice ABH Biozzi	↑mRNA Il-1β, TGFβ and COX2 after 24h	If peripheral LPS: unclear results
				↑Iba-1+ cells and ICAM+ cells after 24h	
Jin et al., 2007	Endogenous murine	MPTP-intoxication-induced murine α-syn aggregation	Prostaglandin E2 receptor subtype2 knockout	Lack of EP2 abolished MPTP-induced ↑ aggregated α-syn	*In vitro* EP2 knockout microglia has ↑ ability to clear α-syn
Miller et al., 2007, Su et al., 2008, 2009	DM A30P-A53T *	Overexpression under TH promoter	Mouse C57/Bl6	↑% of activated Iba-1+ cells in SN↑mRNA TGFβ SN and StrAltered expression on inflammation-related genes2009- ↑number and activated Iba-1 cells early and long-lasting in SN, not str	Microgliosis preceding cell death
Stefanova et al., 2011	WT	Overexpression under PLP promoter x TLR4 knockout	Mouse (hybrid background)	↑ DAergic cell loss and motor defects↑TNF and astrogliosis↓Phagocytosis of α-syn by microglia	*No change in IL-10, IL-6, IL-1α, IFNγ and GM-CSF*
Watson et al., 2012	WT	Overexpression under Thy1 promoter	Mouse hybrid C57/Bl6+DBA2	↑Activated Iba-1+ microglia in str (1m) in SN (22m)	*No change in ctx and cerebellum*
				↑TNF in str (1m) in SN and blood (5–6m)	
				SN ↓TLR1 (1m);↑TLR1,4 and 8 (5-6m);↑TLR2 (14m)	
				↑CD4+ and CD8+ T-cells% in blood (22m)	
Theodore et al., 2008	WT	Local rAAV-A53T-α-syn injection in SN	Mouse C57/Bl6	↑CD68 expression↑TNF, IL-1α, IL-6 and ICAM mRNA at 2w and TNF mRNA at 4 weeks in SNInfiltration of T-cells and B-cells	*No change in the alternative activation markers IL-4, IL-13 and arginase1*
Sanchez-Guajardo et al., 2010	WT	Local rAAV-WT-α-syn injection in SN	Sprague–Dawley Rat	↑Number of Mac1 cells and changes on profile↑In MHCII expression↑CD68 expression if cell death occursInfiltration of T-cells	
Chung et al., 2009	A53T	Local rAAV-A53T-α-syn injection in SN	Sprague–Dawley Rat	↑Levels (WB) of Iba-1 in str not in SN. Activated morphology in Str↑IL-1β, IFNγ and TNF in str not in SN	

In another approach, direct injection of monomeric or oligomeric α-syn into the SN also induced microgliosis which supports the role of α-syn as a direct initiator of inflammation (Wilms et al., 2009; Couch et al., 2011), a concept that will be discussed in more detail below. The use of HA-TAT internalization signal peptide to introduce nitrated α-syn within cells (as opposed to addition of α-syn extracellularly) confirmed that microgliosis correlated with α-syn-induced neurodegeneration (Yu et al., 2010). The involvement and association of both α-syn and microglia in DA neuron cell death has also been proposed in the MPTP neurotoxin model of nigral cell death (Jin et al., 2007).

In agreement with studies involving classic transgenic mouse lines, our studies with rAAV (recombinant adeno-associated virus) vector-mediated α-syn overexpression in rodents or primates suggest that microgliosis is an early event related to the presence of α-syn expression that precedes cell death (Sanchez-Guajardo et al., 2010; Barkholt et al., 2012). Moreover, there appears to be a threshold of WT α-syn expression required to induce PD-like pathology. Once this threshold is reached, DA cell death and increased Mac1+ cells with macrophagic features can be observed by 8 weeks. Animals that do not reach the threshold for DA cell death do show an increase in microglia and MHC II expression, although this peaks at 4 weeks. (Sanchez-Guajardo et al., 2010). The rAAV-driven overexpression of α-syn in mouse in the absence of DA neuron loss led to persistent deposition of IgG and altered expression of pro-inflammatory cytokines in SN (Theodore et al., 2008). In most cases, the microgliosis was mainly observed in SN and to a lesser extent in striatum. However, one group reported that overexpression of A53T in rats by rAAV induced Iba1+microgliosis in striatum with no change in SN at 8 weeks, whereas in previous work the authors reported detectable cell death at 16 weeks (Chung et al., 2009). As expected, they observed an increase in the proinflammatory cytokines TNF, IFNγ and IL-1β in striatum, but not in the SN.

### α-Syn as initiator of microglia activation *in vitro*

The rAAV-mediated α-syn overexpression in primates led to microgliosis in SN that preceded cell death (when WT α-syn was used) and persisted for one year after this was initiated (Barkholt et al., 2012). Although the presence of activated microglia following the onset of cell death is a well-accepted notion, the fact that microglia is activated early in α-syn models and in patients suggests that α-syn-related events involved in the neurodegenerative process that occurs prior to cell death are capable of activating the microglia. In this respect, α-syn as well as CM (conditioned media) from α-syn expressing neurons (that could include other proteins besides α-syn) has been shown to robustly activate microglia *in vitro* in a number of independent studies. Therefore the release of α-syn from cells is a phenomenon that may be required for the suggested prion-like spread of α-syn pathology (for a review see Steiner et al., 2011). This will lead to the presence of α-syn extracellularly that can then be taken up by microglia and the efficiency of this process seems to depend on the activation state of the microglia and on whether α-syn is in monomeric or oligomeric form (Lee et al., 2008; Park et al., 2008).

Multiple independent laboratories have reported on the effects of extracellular α-syn on microglia and it appears that the effects differ depending on: (i) origin (CM, recombinant purified protein, etc.), (ii) type (WT or mutant), (iii) molecular state (monomeric, oligomeric or filamentous), (iv) post-translational modification (nitration or nitrosylation) of α-syn and (v) whether a cell line or primary microglia are used (Table 2). BV2 is a well characterized and widely used microglia cell line because of its close resemblance to primary brain microglia (Blasi et al., 1990). However, responses in this cell line often differ from the observed responses in both primary and *in vivo* microglia (Henn et al., 2009). Incubation of BV2 cells with exogenous non-aggregated α-syn has been reported to lead to increased phagocytosis (Park et al., 2008), increased TNF synthesis (Alvarez-Erviti et al., 2011), NFκB p65 nuclear translocation (Couch et al., 2011) and migration (Kim et al., 2009). Debris from α-syn transfected astrogliomas that presented amorphous α-syn inclusions (Stefanova et al., 2002) induced phagocytosis of α-syn in BV2 cells through a TLR4-dependent mechanism (Stefanova et al., 2011). However, the incubation of fibrillar α-syn abolished the α-syn-induced phagocytosis and reduced the overall phagocytic ability of the BV2 cells (Park et al., 2008). These interesting findings suggest that certain molecular species of α-syn may interfere with the phagocytic ability of microglia. Indeed, the overexpression of α-syn reduced LAMP1 (lysosome-associated membrane protein 1) and BV2 phagocytic ability even as their pro-inflammatory profile persisted, as evidenced by increased TNF secretion, COX-2 expression and NO (nitric oxide) production (Beraud et al., 2011; Rojanathammanee et al., 2011).

**Table 2:** *In vitro* studies of effect of α-synuclein on microglia α-syn, α-Synuclein; O, oligomeric; F, fibrils; PF, protofibrils; N, nitrated; CM, conditioned media; ROS, reactive oxygen species; KO, knockout; *DM, double mutant not occurring in humans; ** α-syn content in CSF is not addressed; in italics, markers included in the studies that did not show any change.

Reference	α-Syn	Type/origin	Cell	Observations	Notes
Zhang et al., 2005	WT	7-days aged O-α-syn	1. Rat mesencephalic neuro-glia	↑ROS and PGE2	These changes required phagocytocis of α-syn. Mediated by (but not only) NADPH oxidase
			2. Rat primary microglia	Activated microglia profile	
				*No change in TNF or nitrites*	
Jin et al., 2007	WT	1. LB disease *post mortem* brain tissue	Prostaglandin E2 receptor subtype2 KO primary microglia	1. Lack of EP2 ↑ α-syn clearance	
		2. 7-days aged O-α-syn		2. O-α-syn induced p67 and p47 phox translocation	
				P47 translocation is EP2 dependent	
Thomas et al., 2007	Murine	Non-aggregated and aggregated (O,F and PF) N-α-syn 50–500 nM	Mouse primary microglia	↑ROS production if α-syn aggregated. Inhibited by K^+^ or H^+^ channels blockers	↑ ROS not due to debris presence or unspecific amyloid effect.
Zhang et al., 2007	WT; A53T; A30P	Recombinant 250 nM	Rat midbrain neuroglia	DAergic toxicity in all three α-syn mediated by microglia production of superoxide and intracellular ROS. This is partially Mac-1 mediated and independent of phagocytosis	Mediated by (but not only) *Phox*
Su et al., 2008	WT	Recombinant 10, 50 and 250 nM	Mouse primary microglia	↑Activated Iba1+ cells↑TNF release↑mRNA TNF, COX2, IL-1β, IL6, NOX and iNOS↑ROS	This is attenuated in CD36 KO cultures and mediated by ERK1/2 phosphorylation
Su et al., 2009	(*)DM- A30P–A53T	Recombinant 2.5, 5 and 10 nM	Mouse primary microglia	↑Activated Iba1+ cells↑mRNA TNF, IL-1β, IL6, IL10, COX2, NOX2 and iNOS↑TNF and IL-1β	This is attenuated in CD36 KO cultures and mediated by ERK1/2 phosphorylation
Klegeris et al., 2008	WT; A53T; A30P; E46K; Δ71–82	Recombinant monomeric	1. Human THP-1.	1. ↑TNF release by A53T and IL-1β by A53T an A30P in naïve THP-1. CM from IFN-γ primed-THP-1 exposed to any α-syn induced SHSY5Y toxicity and ↑ TNF and IL-1β release	Analysis of phosphorylation in WT α-syn-primed THP-1 showed ERK1 and 2, p38 and JNK MAP kinases activation
			2. Human primary microglia	2. CM from WT IFN-γ-primed-microglia induced toxicity SHSY5Y	
Park et al., 2008	WT; A53T; A30P; E46K; Δ1-95; NAC	Aggregated (F) and monomeric	1. BV2.	1. All monomeric α-syn and Δ1-95↑phagocyt Aggregated α-syn ↓phagocyt and abolish monomeric induced phagocytosis.	↑Phagocyt not mediated by CR3, α6β1 integrin or CD47. β and γ-syn did not ↑phagocytosis
			2. Rat primary microglia	2 and 3. Monomeric α-syn ↑phagocyt	
			3. RAW 264.7 murine macrophage		
Reynolds et al., 2008a	Murine	Aggregated (O) unmodified and N-α-syn 100 nM	1. Mouse primary microglia.	*N*-α-syn ↑ TNF, IL-6, MCP-1 and IFN-γ	GDNF and BDNF is also increase in microglia upon stimulation
			2. (1) co-culture with MES23.5	Both unmodified and N-α-syn induced TH+ cell death in co-culture or using CM from (1)	Transcriptome analysis, please refer to the original article.
				↑ mRNA in microglia: TNF, CCl2, IL-6, IL-1β, NFkB1-2, Rela, Fos, Raf1, Card10 and Casp8	
Reynolds et al., 2008b	Murine	Aggregated *N*-α-syn 100 nM	Mouse primary microglia	Complex response of microglia with both proinflammatory and putative neuroprotective profile	If unaggregated N-α-syn did not induce changes.
Lee et al., 2009	WT; A53T; A30P; E46K	Recombinant monomeric 0.1, 1, 5 and 10 μM	1. Macrophages RAW264.7	1. WT ↑TNF, COX2 and iNOS. All mutants ↑TNF	Same was true for β and γ-syn NAC sequence is not necessary for activation
			2. Human primary macrophages	2. WT ↑TNF	
Wilms et al., 2009	WT	Recombinant α-syn O and F 0.5, 5, 50 and 500 ng/ml	Rat primary microglia	O-α-syn most efficiently induced ameboid shape and ↑NF-κB, p38 and ERk1/2 MAP kinases and ↑nitrites production	
Kim et al., 2009	WT; A53T; A30P	1. Transient transfection	1. BV2.	↑CD44 expression and cleavage ↑Mt1-MMP expression by BV2 transfected and exogenous α-syn on BV2 and microglia	If transplanted *in vivo* α-syn pretreated BV2 migrates from striatum to SN in a 6-OHDA PD model
		2. Recombinant 100 nM	2. Mouse primary microglia	A53T overexpression ↑BV2 migration	
				α-Syn overexpression in BV2 ↑ERK1/2	
Roodveldt et al., 2010	WT; A53T; A30P; E46K	Monomeric (0.2, 1 and 5 μg/ml)	1. Mouse primary mixed glia2. Mouse primary microglia	↑IL-6 Mixed all α-syn. Enrich. A30P and E46K↑IL-1β A30P and E46K↑IL-10 Mixed A30P;↓IL-10 Enrich Microg A53T↑IL-10, RANTES, MCP-1 and MIP-1α: A30P and E46K↑ MCP-1 and MIP-1α: Mixed WT↑ TNF and IFNγ Enrich A30P↑Phagocyt. WT and A53T; ↓Phagocyt. A30P and E46K	
Lee et al., 2010	WT; A53T	1. Monomeric 1, 5, 10μM.	Rat primary microglia	1. ↑TNF, Nitrite, IL-1β and ROS; ↑NFkB and AP-1 DNA binding and MAPK phosphorylation	Inhibition of MMP-3, 8 or 9 suppresses pro-inflammatory α-syn effect
		2. CM SHSY5Y-expressing α-syn		2. CM: ↑TNF, Nitrite, IL-1β and ROS	
Schiess et al., 2010	WT	CSF from sporadic PD (**)	HTB15 human glioblastoma	↓ growth rate↑ intracellular α-syn	Unclear whether the increase is due to of ↑ α-syn expression or ↑uptake of exogenous α-syn
Alvarez-Erviti et al., 2011	WT; A53T	CM SHSY5Y-expressing α-syn	BV2	CM Wt: N.Ch.	If MPP+ pre-treated SHSY5Y: CM WT: mRNA TNF and IL-1α; ↑TNF
				CM A53T: mRNA Il-1β; ↑IL-1β	A53T: mRNA IL-1β; ↑IL-1β
				Recomb WT: mRNA TNF, mRNA IL-1α; ↑TNF	
Couch et al., 2011	WT	Recombinant	BV2	Translocation NF-κB p65 to nucleus	
				↑TNF release	
Beraud et al., 2011	WT	7-days aged O+F 50 nM	1. BV2	1. ↑NO and TNF release and ↑mRNA IL-1β, Peroxiredoxine-1, Heme oxigenase-1, TLR2 and3 ↓mRNA TLR7	Microglia is activated through a classical activation pathway.
			2. Mouse primary microglia	2. ↑mRNA TLR2, 3, 1 and 7, MYD88, Iba1, NFkB, TNF and IL-1β; ↓mRNA TLR4, 6 and 9 and CD36	
Stefanova et al., 2011	WT	1. Debris from U373 cells (human astrocytoma) transfected with α-syn	BV2	TLR4-dependent phagocytosis	
		2. Recombinant			
Rojanathammanee et al., 2011	WT; A53T; A30P	Transient transfection	BV2	↑COX2↓Phagocytosis and LAMP1↑TNF releaseA53T ↑nitrite and IL6 release	It did not change BV2 survival or lead to neuronal toxicity in co-culture
				*No change in cPLA2, PLD1-2 and COX1*	

Several independent investigators have reported that addition of recombinant monomeric α-syn to rodent primary microglia consistently induces an activated pro-inflammatory microglia profile, including increased expression of TNF, IL-1β, IL-6, COX2 and iNOS (Su et al., 2008, 2009; Lee et al., 2010a) and this profile was also true when using cells of human origin (Klegeris et al., 2008; Lee et al., 2009b). Finally, differences between WT α-syn and α-syn mutant proteins in their ability to activate glia have been highlighted recently and are improving our understanding of how these mutant proteins might promote inflammatory responses in the familial forms of PD (Roodveldt et al., 2010).

Activation of microglia by α-syn is sufficient in culture to induce DA cell death via ROS production (Zhang et al., 2007), and production of TNF by microglia has been shown in turn to promote cell death in α-syn-expressing neurons (Stefanova et al., 2003), further supporting the potential deleterious role of persistent activation of microglia on DA neuron survival. Consistent with this idea, microglia activation leading to macrophagic features has been shown to lead to NO production that in turn can induce nitration of α-syn in neighbouring neurons and result in cell death (Shavali et al., 2006). Accordingly intracerebral LPS injections in PrP WT or A53T α-syn transgenic mice resulted in free radical formation (most likely from microglia origin), with subsequent nitration, aggregation of α-syn and DA neurodegeneration (Gao et al., 2008). However, α-syn-induced microglia activation can also promote the expression of neuroprotective growth factors such as BDNF (brain-derived neurotrophic factor) and GDNF (Reynolds et al., 2008a). ROS-producing microglia are likely to be functionally associated with cell death, whereas growth factor-expressing microglia are likely to be functionally associated with neuronal repair and survival (Sawada et al., 2006). Taken together, these observations strongly support a dual role for activated microglia in human brains with ongoing synucleinopathies. Indeed, it has been proposed that in neurodegenerative conditions like AD, microglia have two stages of activation: M1 and M2 (for a review see Varnum and Ikezu, 2012). M1 is associated with classical TNF/IFNγ-mediated pro-inflammatory activation; whereas the alternatively activated M2 is subdivided into the M2a stage characterized by alternative activation-anti-inflammatory cytokines (IL-4 or IL-13) and the M2c stage as the deactivation-wound healing stage with cytokines that promote tissue repair [IL-10 and TGFβ (tumour growth factor β)]. One could likewise propose the involvement of all three types of microglia activation stages in PD based on the reported increase in all these cytokines and of the microglia features observed (Mogi et al., 1996; Brodacki et al., 2008). We propose that, as has been proposed for AD, during PD progression a shift occurs from an M2 to an M1 phenotype due to different signals and cues in the local environment (which may include the molecular form of α-syn, neuronal activity, peripheral cells infiltration etc.). Therefore future immunomodulatory therapies in PD should focus on promoting the M2 profile over the M1 profile in order to promote regeneration and neuroprotection.

### α-Syn function in microglia

Despite the fact that α-syn was originally believed to be functionally important only in neurons, there are now several studies demonstrating a role for endogenous α-syn in microglia function. For instance, α-syn appears to take part in the normal/homoeostatic microglia activation, as microglia of mice lacking SNCA exhibited a proinflammatory profile and reduced phagocytic activity (Austin et al., 2006, 2011). This alteration has been proposed to be related to the suggested role of α-syn in lipid-mediated signalling via changes in phospholipase, an important molecule in many events related to activation, phagocytosis and synthesis of inflammatory molecules in macrophages (for review see Golovko et al., 2009). Therefore a change in the availability of α-syn in microglia may lead to an inefficient response to external signals that in turn affect neuronal survival.

The ongoing debate in the PD field as to whether disease results from a loss- or a gain-of-α-syn-function may also be applicable to the microglia population. To investigate this possibility different groups have used transient overexpression of α-syn in microglia to further understand the role of α-syn in this population. BV2 cells overexpressing WT, A53T or A30P α-syn displayed increased COX-2 levels and TNF secretion and impaired phagocytic activity as shown by decreased LAMP 1 (Rojanathammanee et al., 2011). This suggests that a lack or mishandling of α-syn can weaken the ability of microglia to clear the microenvironment by impairing phagocytosis. In addition, BV2 overexpressing A53T α-syn increased nitrite production and IL-6 secretion, thereby contributing to an enhanced pro-inflammatory environment (Rojanathammanee et al., 2011). Finally, the expression of α-syn in microglia has been shown to promote their migratory ability by increasing the expression of CD44 and the cell surface protease membrane-type 1 MMP (matrix metalloproteinase) (Kim et al., 2009).

α-Syn-induced microglia activation is likely to result in activation of multiple intracellular signalling pathways. It has been shown that MMPs and PAR-1 (protease-activated receptor 1) are involved in the cellular activation process initiated by α-syn (Lee et al., 2010a). Activation of ERK1/2 (extracellular-signal-regulated kinase 1/2) and p38 MAPK (mitogen-activated protein kinase) has been associated with exposure to both oligomeric and monomeric α-syn (Su et al., 2008, 2009, Wilms et al., 2009) and so has activation of membrane receptors, including CD36 (Su et al., 2008, 2009; Wilms et al., 2009; Beraud et al., 2011) and TLR (Beraud et al., 2011; Stefanova et al., 2011). NFκB has been consistently implicated in studies with monomeric, oligomeric, aggregated or nitrated α-syn in rodent cell lines and human microglia (Klegeris et al., 2008; Reynolds et al., 2008b; Wilms et al., 2009; Lee et al., 2010a; Couch et al., 2011). As far as the contribution to degeneration from the different glia populations, one report demonstrated that astroglia in culture can protect DA neurons against α-syn-induced toxicity, whereas the percentage of microglia in the culture was directly correlated to neuron cell death (Zhang et al., 2005). In support of this idea, neuronal survival outcomes differ when microglia are placed in culture alone compared with in culture with a mixed glia population, suggesting a modulatory role for astroglia (Roodveldt et al., 2010).

α-Syn is also expressed by a number of peripheral immune cells, including T-cells, B cells, NK (natural killer) cells and monocytes (Shin et al., 2000). Furthermore, the level of α-syn increases in macrophages and lympocytes upon LPS activation, suggesting a regulatory role for the protein in these cells (Tanji et al., 2002; Sergeyeva and Sergeyev, 2011). Interestingly autologous transfer of LPS-activated macrophages increased levels of circulating anti-α-syn antibodies and resulted in accumulation of endogenous α-syn in DA neurons, highlighting the cross-talk between the CNS and the periphery (Sergeyeva and Sergeyev, 2011). Indeed, the synergistic effect of peripheral inflammation and α-syn toxicity in the brain was recently demonstrated by the effects of a single peripheral LPS injection in α-syn transgenic mice. These animals developed persistent inflammation, and via free radicals, likely from activated microglia, induced nitration and aggregation of α-syn, resulting in DA neuron degeneration (Gao et al., 2011). Taken together, these findings underscore the potential relevance that seemingly unrelated peripheral immunological events could have on the onset and/or progression of disease in patients with PD.

## MICROGLIA AS THE ORCHESTRATORS OF NEUROIMMUNITY

### Microglia activation pattern during PD: MHC II compared with CD68

Microglia activation is an early event that progresses, in nature and features, as PD pathology develops and results in cell death. This raises the question as to whether microglia activation is detrimental at all stages of the disease or whether some inflammatory processes may be acting to limit injury and promote tissue repair. Microglia express many different receptors and surface proteins that allow them to undertake many different, and in some cases opposing, functions. In animal models of PD, the two most studied microglia proteins are MHC II, which denotes antigen presentation ability, and the macrophage–myeloid-associated antigen CD68 which is present on phagocytic microglia.

The expression of CD68 in post-mortem SN of PD patients has been correlated to disease duration (Croisier et al., 2005). In contrast, HLA–DR expression on microglia in post-mortem human SN from PD patients correlated positively with α-syn deposition, but did not correlate with clinical disease severity or disease progression (Croisier et al., 2005). In agreement with this finding, the number of HLA–DR immunoreactive microglia over the disease course or in patients with longer duration of disease did not seem to change (Orr et al., 2005). Interestingly, in that same study double-labelling immunofluorescence revealed that staining for IgG colocalized with α-syn in pigmented SN neurons and significantly more IgG immunopositive neurons were detected in early-stage compared with late-stage PD. These observations were interpreted to suggest that IgG coating of melanized SN neurons occurs early in the disease prior to gross degeneration and may be a process that drives the disease phenotype by triggering complement activation or targeted attack by surrounding microglia expressing Fc receptors. GWAS (genome-wide association studies) found significant associations between specific SNPs in the HLA gene loci and risk for late-onset PD (Hamza et al., 2010; Nalls et al., 2011), but it is not yet known how these variants affect HLA–DR expression on microglia or other APCs (antigen-presenting cells) in the CNS or how they affect disease severity or progression.

It has been previously postulated that the differential expression of CD68 compared with MHC II on microglia could signal distinct polarization pathways resulting in detrimental compared with beneficial microglia (Table 3). Specifically CD68 is selectively expressed when rAAV-hα-syn-induced TH+ cell death occurs in SN, peaks in expression after cell death has taken place, and is down-regulated afterwards (Sanchez-Guajardo et al., 2010). Similarly, 6-OHDA medial forebrain bundle lesions in rats have been reported to induce up-regulation of CD68 expression in SN starting at day 3 and achieving maximal expression at day 14 after the significant TH+ cell death has occurred (Marinova-Mutafchieva et al., 2009). Henry et al. (2009) monitored morphological changes in microglia (i.e. cells with enlarged soma and short un-ramified processes were considered activated) and found that the peak of microglia activation occurred 6 days after 50% of all TH+ neurons had been lost. Other groups who have measured markers of neuroinflammation in mouse SN after injections of rAAV-encoding human (h)α-syn found that microglia transiently up-regulated CD68 4 weeks after α-syn overexpression and down-regulated it by 12 weeks, which also coincided with detectable loss of TH+ neurons (Theodore et al., 2008). On the other hand, MHC II expression has been reported to peak earlier than CD68 and to persist longer (Akiyama and McGeer, 1989; Marinova-Mutafchieva et al., 2009; Vazquez-Claverie et al., 2009; Sanchez-Guajardo et al., 2010), in some cases reaching higher expression levels when cell death does not take place (Vazquez-Claverie et al., 2009; Sanchez-Guajardo et al., 2010; Barkholt et al., 2012). In the Thy1 α-syn transgenic mouse line, microglia up-regulate MHC II in SN after 14 months, when α-syn expression reaches a 2-fold increase compared with the WT endogenous levels (Chesselet et al., 2012; Watson et al., 2012). Similarly, following a 6-OHDA lesion in the rat medial forebrain bundle, microglia with amoeboid morphology appeared at day 1, and by day 7 a large number of MHC II+ cells and CD68+ cells were observed in close contact with healthy DA neurons whereas only CD68+ cells were found in close proximity with caspase+ DA neurons and attached to degenerating axons and dendrites of DA neurons in SN; this was followed by the progressive loss of TH+ cells that peaked (51%) 9 days after lesion (Marinova-Mutafchieva et al., 2009). These data suggest that the activation of microglia precedes the peak of DA neuron cell loss and that neurons undergoing degeneration may be phagocytosed prematurely by phagocytic microglia.

**Table 3:** Microglia activation and neuroinflammation in rodent models of nigral dopaminergic cell death MPTP, 1-methyl-4-phenyl-1,2,3,6-tetrahydropyridine, 6-OHDA, 6-hydroxi-dopamine; AAV, adeno associated virus; MHC I/II, major histocompatibility complex I/II; MFB: medial forbrain bundle; TLR, Toll-like-receptor; TH, thyrosine hydroxylase; DA, dopaminergic; SN, substantia nigra; Str, striatum; α-Syn, α-synuclein; WT, wild-type; Tg, transgenic; mo, months; wks, weeks; d, day(s); hr, hour(s); nd, not determined.

Model	Species	TH+cell loss in SN	TH+fibre loss in Str	Gliosis in SN	MHC II	CD68	Comment	Reference
Subchronic MPTP	Mouse	2 hr peak d7	2 hr recovered by d28	Peak at d1, persists at lower levels to d21	2 hr	nd	C1q expression (after 2 hr)	Depboylu et al., 2011
Intravenous MPTP, chronic vs subacute	Cynomolgus monkey	Subacute <acute	nd	nd	all positive; peak at 6 mo for chronic and persists to 35 mo	nd	Vazquez-Claverie et al., 2009)	
Unilateral intra-SN delivery of two different dose of AAV2/4-αSyn	Rat	Only at high α-syn dose (4 wks)	Progressive in both groups high >> low dose	Neurodegeneration of greater magnitude; peak at 4 wks and prior to cell death (8 wks)	Neurodegeneration > cell death; peak at 4 wks, persists to 15 wks	Only cell death, at all times, peak 8 wks	T cells and B cell infiltration, neurodegeneration; MHC II+ in Str	Sanchez-Guajardo et al., 2010
Unilateral intra-SN delivery of AAV2/5-WT or A53T α Syn	Marmoset monkey	A53T α-syn after 12 mo	nd	WT 100% increase A53T 80% increase	A53T > WT	nd	HLA-DR+ CD19+ cells; 4 morphologies, A53T more polarized than WT	Barkholt et al., 2012
Unilateral intra-SN delivery of AAV2-α-Syn	Mouse	nd	nd	nd	nd	4 wks, 2 and 12 mo	stereologic quantification of lymphocytes; IgG deposition	Theodore et al., 2008
Unilateral injection 6-OHDA in MFB	Rat	From d3 peak d9	nd	From d1; peak d15	From d1; peak at d9 (>CD68); persists to d15	From d3 peak at d15 (>MHC II)	MHC II in contact with neurites or live cells; CD68 in contact with caspase+ cells	Marinova-Mutafchieva et al., 2009
Unilateral injection 6-OHDA in nigrostriatal system	Rat	50% d1	nd	From d5 activated; peak at d7–14 and persists to d35	nd	nd	Two morphologically distinct microglia; activated: big stoma, short processes	Henry et al., 2009
Unilateral injection 6-OHDA in nigrostriatal system	rat	nd	nd	nd	from d4-6, d6< MHC I d30 present (no MHC I); d90 absent	nd	MHC I: d3 look like leucocytes, d4–6 microglia	Akiyama and McGeer, 1989
Intranasal injection of 6-OHDA	Rat	50%	75%	Yes	nd	nd	Preceded onset of dopaminergic loss	Armentero et al., 2006
Transgenic α-Syn overexpression	Tg mice (Thy1 promoter)	Non	Non	From 1mo in Str; from 5-6mo in SN	After 14mo in Str	nd	Gliosis and MHC II expression independent of DA cell loss; T cells ↑in serum (22 mo); Altered TLR expression	Watson et al., 2012

Taken together, these data strongly suggest that microglia acquire different phenotypes during the progression of PD. We propose that during the early stages of PD and prior to nigral cell death, microglia up-regulate MHC II, which may be reflective of presentation of self or foreign antigens to tissue-specific T-cells or be an adaptive and potentially beneficial immune response. On the other hand, M1 CD68+ microglia observed during the later stages of PD display highly phagocytic activity that likely contributes to DA neuron loss. This pattern appears to be consistent across species (monkey, rats, mice) and models (α-syn, MPTP, 6-OHDA). More widespread use of non-invasive imaging technologies with ligands specific for the various activation states of microglia subpopulations will be needed to nail down the kinetics of such a process in various stages of PD.

### Microglia and the adaptive immune response: cross-regulation and/or induction?

T lymphocytes are activated through their TCR (T-cell receptor) by recognizing cognate antigen on MHC molecules and by receiving co-stimulation and appropriate cytokine signalling from APCs. Normally this takes place in the lymph nodes or germinal centres in the spleen, but it is known to happen *in situ* in other tissues under special conditions. Regardless of the location, the ensuing response needs to be sustained through TCR/MHC contacts for it to be effective until memory T cells are generated (Freitas and Rocha, 1999). Microglia, although normally associated with phagocytic functions and viewed primarily to be a brain macrophage, have the ability to act as an APC. Indeed, *in vitro* studies with primary mouse microglia clearly demonstrate that microglia can present antigen via MHC II and activate CD4+ T-cells to differentiate down various lineages depending on the experimental conditions (Fischer et al., 1993a; Carson et al., 1999; Re et al., 2002). When stimulated with GM-CSF (granulocyte/macrophage colony-stimulating factor), mouse primary microglia have been shown to induce Th1 (pro-inflammatory) and Th2 (anti-inflammatory) T-cell proliferation and cytokine production in an IFNγ-independent manner (Fischer et al., 1993b). When the microglia are activated with LPS and IFNγ, however, T-cells differentiated into Th1 cells but did not proliferate (Carson et al., 1999). These findings suggest that the phenotype and effector functions of microglia are dependent on the specific signalling factors presented to them; and perhaps more importantly that microglia are capable of activating and sustaining an adaptive immune response in brain parenchyma.

CD8+ T-cells, also known as cytotoxic lymphocytes, are activated by MHC I, which is composed of two chains, one of which is β2-microglobulin (Bjorkman and Parham, 1990). Although β2-microglobulin has been specifically observed in the striatum of PD brains (Mogi et al., 1995), its role in PD progression is not known. MHC I is expressed by all cells in an organism at a basal level, but unless the antigen they present induces binding of a CD8+ T-cell, the peptide will dissociate from the MHC I complex and the complex will be internalized. Thus, MHC I can usually only be detected after TCR recognition (York and Rock, 1996). In animal models of PD, it has been observed that nigrostriatal 6-OHDA lesions are associated with the appearance of round MHC I+ cells on day 3 and MHC I+ microglia peak between days 4 and 6, after which the expression of MHC I declines and is undetectable after day 30 (Akiyama and McGeer, 1989). These observations indicate that cytotoxic T-cells invade injured brain parenchyma in rodent models of nigral cell death. The CD8+ cytotoxic T-cell response can be sustained/enhanced by interacting with Th cells, a requirement for CD8 memory to be induced (Zaragoza et al., 2011). In support of this, absence of CD4+ cells reduced the number of CD8+ T-cells present in SN after MPTP intoxication of CD4-deficient mice (Brochard et al., 2009).

### Lymphocytes in PD: how to tip the balance towards protection?

Recruitment of lymphocytes to brain parenchyma has been recapitulated in several animal models of PD. T-cell infiltration has been observed as an early event preceding CD68 expression when α-syn is overexpressed via rAAV in mouse SN (Theodore et al., 2008). The type of T-cell response appears to vary depending upon whether cell death was observed: in its absence, the response was primarily composed of CD4+ T-cells, whereas when cell death occurred T-cell infiltration was delayed and the ratio CD4+ T-cells compared with CD8+ T-cells was decreased (Sanchez-Guajardo et al., 2010). In the MPTP model, CD4+ T-cell infiltration peaked before that of CD8+ cells, but CD8+ cells were more numerous than CD4+ T-cells (Brochard et al., 2009). While in the rAAV–hα-syn model the T-cell response correlated with the peak of microgliosis, in the MPTP model T-cells infiltrated the brain after microglia cell numbers peaked and cell death had occurred. Given that the MPTP model induces more acute nigral cell death relative to the slower degeneration observed with the rAAV–α-syn overexpression model, the differences observed between the models may reflect the fact that molecular processes are happening at different time points relative to progression of nigral cell death. In both studies, however, minimal infiltration of T-cells into the striatum was observed, suggesting that T-cells are homing to sites where cell death is or will be occurring, rather than to the DA terminals.

Further support for a role of T-cells in PD-like nigral degeneration comes from studies in mice with severe combined immunodeficiency that lack T-cells. These mice have been reported to be relatively resistant to MPTP intoxication and transfer of T-cells from nitrated α-syn-immunized mice accelerated MPTP-driven neurodegeneration (Benner et al., 2008). Brochard et al. (2009) further dissected the immune response by using TCRβ-, CD4- and CD8-deficient mice as MPTP recipients, and found that the transfer of CD4+ T-cells accelerated nigral degeneration, whereas CD8+ T-cells did not appear to play a significant role (Brochard et al., 2009).

A remaining question is how T-cells contribute to the neurodegenerative process. Do they directly induce neuronal death, for example, via Fas signalling (Giuliani et al., 2003; Brochard et al., 2009)? Do they change the cytokine microenvironment to polarize glia or signal to neurons (Kebir et al., 2007; Mount et al., 2007)? Or do they interact with glia in a cell-to-cell contact-dependent manner? Some insight into how T-cells and microglia interact has been gained in the last few years (for a review see Appel et al., 2010). *In vitro* studies where CD4+ CD25+ and CD4+ CD25- T-cells were co-cultured with microglia previously activated with nitrated α-syn showed that T-cells modulated microglial phenotype. Specifically, CD4+CD25+ T-cells suppressed ROS production and NFκB activation while CD4+CD25- T-cells potentiated the neuroinflammatory response (Reynolds et al., 2009a, 2009b). Vaccination of rats with α-syn has been shown to alter microglial morphology, increase proliferation and induce CD68/CD4/MHC II/MHC I expression. These specific immune changes occurred prior to robust overexpression of α-syn within the nigrostraital system and resulted in a marked reduction of α-syn-induced striatal pathology and correlated with CD4+ T-cell infiltration (Sanchez-Guajardo et al., 2013). All these observations strongly suggest that interactions between T-cells and microglia have the potential to modify cellular phenotype in either direction and that CD4+ T-cells can modify the fate of microglia as much as microglia can activate and differentiate CD4+ T-cells along various lineages. Understanding this balance could give insight into how to tip the balance of adaptive immune responses towards a more protective response during PD.

Several studies support a role for adaptive immune responses in nigrostriatal degeneration. For instance, IgG immunoreactivity (Theodore et al., 2008; Sanchez-Guajardo et al., 2013 and B cell infiltration (Sanchez-Guajardo et al., 2010; Barkholt et al., 2012) have been observed within the nigrostriatal pathway when α-syn is overexpressed in animals. Other researchers have shown that IgG serum transferred from PD patients to mouse SN, was also able to elicit TH+ cell death (Chen et al., 1998; He et al., 2002). IgG triggers cell death via several pathways. Studies with FcgRI/III-deficient mice and Fab fragments have shown protection against human IgG-induced cell death (He et al., 2002). In the SN of PD patients, FcgRI (CD64)/FceRII (CD23) have been observed on microglia and FcgRIII (CD16) on cells that morphologically resembled lymphocytes (Hunot et al., 1999; Orr et al., 2005). NK cells also express FcR and kill cells with FcR-bound IgG. Notably, this antibody-mediated cell-dependent toxicity is increased in PD (Bokor et al., 1993). Finally, γδT CD4 cells mediate the humoral response against neurons via increased hsp65/70 (Fiszer et al., 1996). Besides binding Fc receptors, IgG can activate the complement pathway *in vitro*. As evidence for this, PD patient IgG and hrC5a (human recombinant complement 5a) induced neurotoxicity in mixed neuron–glia co-cultures (Wang et al., 2007). Similarly, PD patient serum added to dissociated mesencephalic–striatal co-cultures resulted in reduced DA uptake and TH+ cell loss; however, this only occurred when PD patient serum was added together with reconstituted rabbit complement (Defazio et al., 1994). All these studies strongly suggest that the humoral immune response is modulating microglia in PD through FcR and CRs (complement receptors). This modulation may be detrimental as explained above, but as will be discussed below, IgG has also been implicated in α-syn clearance and neuronal protection, further supporting the idea that there is a fine balance between the beneficial and detrimental aspects of the immune response during the nigral degeneration that occurs in PD.

### Microglia as modulators of innate immunity

Microglia mediate the innate immune response triggered by PAMPs (pathogen-associated molecular patterns) by modulating TLRs, scavenger receptors, phagocytosis and complement-mediated responses (reviewed in Saijo and Glass, 2011). Microglia have been reported to express TLR1 through TLR9 proteins (Jack et al., 2005), but such expression is dynamically regulated (reviewed in Lehnardt, 2010). For example, TLR2 is expressed only by activated microglia whereas TLR4 is constitutively expressed at low levels, but can be increased by certain stimuli, including MPTP. The strongest pro-inflammatory response is triggered by activation of TLR3, which leads to high TNF production. TLR1 induces IL-6 and IL-1, and TLR4 has been shown to induce iNOS, IFNα/β, IL-12, IL-1 and IL-6. By contrast, TLR2 stimulation primarily increases the production of the anti-inflammatory cytokine IL-10 (Olson and Miller, 2004; Jack et al., 2005). Studies with ischaemia models have shown that the pre-activation of microglia through agonists of TLR 2, 4 or 9 induces resistance to injury (reviewed in van Noort and Bsibsi, 2009). The role of TLRs in PD is just starting to be analysed, but the available data suggest that they will probably influence α-syn clearance. These data show that transgenic mice overexpressing α-syn and crossed with a TLR4-deficient mouse had increased motor disability, DA neuron death, TNF and α-syn within the SN, suggesting TLR4 is critical for α-syn clearance (Stefanova et al., 2011). In the Thy-1 α-syn transgenic mouse, there is a diminished TLR1 expression at 1 month of age, but increased TLR8 (at 5–6 months) and TLR2 (at 14 months) relative to WT mice (Chesselet et al., 2012; Watson et al., 2012). *In vitro* studies have also demonstrated that monomeric and aggregated α-syn combinations can regulate TLR gene expression in BV2 cells (up-regulation of TLR2 and TLR3, down-regulation of TLR7) and primary microglia (up-regulation of TLR1, -2, -3 and -7, down-regulation of TLR4) (Beraud et al., 2011). Whether this interaction is direct or mediated by a receptor associated with TLRs is not yet clear as α-syn has also been shown to bind to the scavenger receptor CD36 and induce microglia activation (Su et al., 2008) and it has been reported that CD36 can mediate TLR signalling via non-canonical ligands (Stewart et al., 2010). A cautionary note to investigators revealed by these studies is that the gene expression outcomes differed depending on whether BV2 microglia or primary microglia was used.

Complement-mediated activation of microglia and its role in PD are also gradually being elucidated. As mentioned above, IgG can activate the complement cascade and activate microglia through CRs, but it is not the only way that complement can act on microglia. One of the canonical markers of microglia, CD11b, mediates phagocytosis of iC3b-opsonized cells when it dimerizes with CD18 to form CR3 (Mac1). Within the context of microglia, the complement cascade has been implicated in eliminating synapses during neurodegeneration (Stevens et al., 2007) and in mediating α-syn activation of microglia through Phox (Zhang et al., 2007). Diverse results on the role of C1q in nigral degeneration have been observed. Subchronic administration of MPTP to mice induced the up-regulation of C1q but its expression had no effect on the neurodegenerative process (Depboylu et al., 2011a, 2011b). Others have shown that PD brain co-stains for C1/microglia (Iba1) and that C1q positive cells can ingest neuromelanin, suggesting an active role of C1q-mediated clearance of apoptotic neurons and debris (Depboylu et al., 2011a, 2011b). Nevertheless, in post-mortem brain studies, staining for complement components in PD brains has yielded contradictory results (reviewed in Bonifati and Kishore, 2007). Additional studies and improved tissue processing methods will be needed to sort out these differences and gain a clearer understanding of the role of the complement system in PD pathophysiology.

Most experts agree that microglia are virtually indistinguishable from peripheral macrophages; the only difference being a lower expression of the leucocyte tyrosine phosphatase CD45 in the former and expression of CCR2 (CC chemokine receptor 2) in infiltrating macrophages (Mizutani et al., 2012). The difficulties in differentiating between these two cell types have impaired our ability to determine the contribution of infiltrating macrophages to PD pathogenesis based on currently available cellular markers. To answer this question, however, several groups have resorted to reconstitution experiments using BM (bone marrow) chimaeric mice subjected to MPTP intoxication, where the peripheral macrophages of the host are replaced by transgenic GFP^+^ (green fluorescent protein) macrophages from the donor. One study showed transient iNOS+ cell infiltration into the striatum 1–2 days after MPTP intoxication (Kokovay and Cunningham, 2005), whereas the other showed infiltration to the entire nigrostriatal system prior to the onset of DA neuron loss (Rodriguez et al., 2007). The later study further reported that migration was greater into the striatum, that GFP+ cells clustered around blood vessels and that about half of them where CD68+. Rodriguez et al. further showed that GFP+ cells were present even 60 days after the first MPTP dose was given. Although very thorough, the caveat with this elegant study is that the data were presented as a percentage increase and not actual number of GFP+ cells present, so the relative importance of the infiltrating population could have been overestimated. The most important drawback and criticism levied against studies based on BM chimaeras is that the irradiation protocol required to eliminate the host's immune component may alter the permeability of BBB, thus allowing for greater leucocyte infiltration than would otherwise occur in the absence of irradiation. The legitimacy of this criticism and drawback was directly explored in the ALS (amyotrophic lateral sclerosis) rodent model with BM chimaeras obtained by parabiosis (Ajami et al., 2007) and where BM chimaeric mice where irradiated with a head shield to estimate peripheral immune cell infiltration into the CNS in an animal model of AD (Mildner et al., 2007). The results from various BM chimaera studies in different animal models with and without head irradiation have been reviewed and the conclusion is that infiltration is largely an artefact of the increased permeability of the BBB induced by the irradiation when head shields are not used (Ransohoff, 2007). However, none of the models studied were related to PD, so the extent to which macrophage infiltration is an important component of the progressive pathophysiology of this disease is still undetermined. These studies have been conducted in various other animal models of neurodegenerative disease. For example, in a transgenic mouse model of AD where no irradiation took place, it was observed that when TGFβ production was blocked, CD11c+ macrophages migrated to the CNS and mitigated AD-like pathology (Town et al., 2008). In addition, perivascular macrophages (which express CD163) have been reported to increase in numbers in EAE (experimental autoimmune encephalomyelitis), an animal model of multiple sclerosis (Zhang et al., 2011), and appear to be required for regeneration of nerve grafts (Dahlin, 1995) and in the recovery phase of EAE (Almolda et al., 2010). Given that CD163+ macrophages also express antigen recognition and presentation molecules (Fabriek et al., 2005), they could play an important role in relaying the brain immune response to the peripheral immune system. Specifically, they have been shown to migrate to phagocytose dying neurons (Angelov et al., 1996), their presence has been used as a measure of anti-inflammatory cell activity (Jayadev et al., 2011), and they infiltrate the parenchyma during ischaemia (Mu et al., 2011). In essence, because they can migrate to secondary lymphoid organs, CD163+ cells may be an important link between innate immune system responses triggered by the neurodegenerative process in the brain of PD patients and the induction of the adaptive immune system response in the periphery.

## IMMUNOMODULATION AS A THERAPY FOR PD

The emerging picture of how the immune system (both peripheral immune cells and brain resident microglia) responds and is affected during PD progression has led investigators to explore ways in which modulation of neuroinflammation can rescue from PD-like neurodegeneration in animal models [see Polazzi and Contestabile (2002); Lucin and Wyss-Coray (2009) for review]. In general, researchers have tried to block the effects of microglia-derived inflammatory mediators (Lee et al., 2009a) or modulate the peripheral immune system (Appel et al., 2010; Ha et al., 2012) (see Table 4). We postulate that interventions aimed at blocking microglia-derived inflammatory mediators will most likely be successful in attenuating neuroinflammation and therefore ongoing inflammatory-induced degeneration. We further propose that modulation of peripheral immune cells will ultimately be required to prevent or halt PD.

**Table 4:** Targeted immunomodulatory interventions in pre-clinical models of nigral cell death and their effects on PD-relevant outcomemeasures MPTP, 1-methyl-4-phenyl-1,2,3,6-tetrahydropyridine, 6-OHDA, 6-hydroxi-dopamine; AAV, adeno associated virus; LPS, lipopolysaccharide; CFA, complete Freund's adjuvant; iFA, incomplete Freund's adjuvant; MHC II, Major Histocompatibility Complex II; TH, Tyrosine Hydroxylase; Th, T helper CD4+ cell; Treg, regulatory CD4+ T cell; DA, dopamine; L-DOPA, L-3,4-dihydroxyphenylalanine; dnTNF, dominant negative Tumor Necrosis Factor; GDNF, glia cell line derived neurotrophic factor; TGFβ, transforming growth factor β; PDGF-β, platelet derived growth factor β; NO, nitric oxide; iNOS, inducible nitric oxide synthase; NADPH, nicotinamide adenine dinucleotide phosphate-oxidase; COX-2, prostaglandin-endoperoxidase synthase 2; SN, substantia nigra; SNpc, substantia nigra pars compacta; Str, striatum; α-Syn, α-synuclein; N- α-Syn, Nitrated- α-synuclein; WT, wild-type; wks, weeks; d, day(s); hr, hour(s).

	Model	Target	Effect	Reference(s)
**Intervention (gen therapy)**				
dnTNF	6-OHDA	Soluble TNF	Rescue DA neurons	McCoy et al., 2006, 2008; Harms et al., 2011
GDNF (striatal)	MPTP / 6-OHDA	Neurons	Rescue DA neurons	Lo Bianco et al., 2004; Lindvall and Wahlberg, 2008
GDNF (nigral)	AAV-αsyn overexpression	Neurons	No effect	Lo Bianco et al., 2004; Decressac et al., 2011
**Chemical inhibition of microglia**				
Celecoxib	Striatal 6-OHDA	COX-2	Decreased microglia activationPrevented neurodegeneration	Sanchez-Pernaute et al., 2004
Hydroden Sulfide L-DOPA	Cell lines untreated rats	Neurons	Increase DA and glutathione in brainDecreased IL-6/TNF and NO by microglia	Lee et al., 2010
Minocycline	MPTP	Microglia	Prevents NADPH activation, IL-1 production	Wu et al., 2002
Minocycline	Cell lines with excitotoxins	Microglia	Reduced NO and IL-1	Tikka et al., 2001
Minocycline	MPTP	Microglia	Reduced microglia activation but increased DA cell loss	Yang et al., 2003
Naloxone	LPS	Microglia	Reduced superoxide	Liu et al., 2000
Naloxone	LPS	Microglia	Prevented DA cell loss	Lu et al., 2000
Dexamethasone	Intranigral LPS	Microglia	Prevented catecholamine, TH activity loss	Castano et al., 2002
**Peripheral immunomodulation**				
Glatiramer acetate (GA) = Copolymer 1 with CFA	MPTP T cell transfer from GA immunized animals	T cells	Attenuation SN cell loss in dose dependent mannerT cell accumulation in SNcGDNF induction from astrocytesReduced microgliosis (Note: serum from the immunized animals was ineffective)	Benner et al., 2004; Laurie et al., 2007
Myelin oligodendrocyte glycoprotein (MOG) with CFA or CFA	MPTP immunization of the animals previous MPTP deliver	Peripheral immune system	Partial rescue of DA neuronsNo effect of MOG administration after MPTPCFA alone was partly protective	Kurkowska-Jastrzebska et al., 2005
Systemic CFA	CFA treatment previous to 6-OHDA	Peripheral immune system	Rescues long term behaviour impairmentInduces GDNF in striatumRescues TH+ cells in SNDecreases CD11b expression. Microglia morphology is changedNo effect on astrogliaModerate, transient pro-inflammatory cytokines	Armentero et al., 2006
Live Bacill Calmett-Guerin (BCG); TH in CFA; Copaxone in CFA; CFA alone	MPTP immunization 10d prior	Peripheral immune system	CFA had greater effect than TH or CopaxoneBCG related to *Mycobacterium Tuberculosis* in CFAReduce microgliosisRescued DA transporter loss and DA content in Strslight DA neuron rescue	Yong et al., 2011
Anti-CD3 activated CD4+CD25+ T cells	Adoptive transfer 12hr after last MPTP dose	Microglia	Changes in CD11b reactivityIncreased IL-10 and TGFβ in midbrainDecreased iNOS and TNF in midbrainIncreased TH+ neuron survival	Reynolds et al., 2007
Treg vasoactive intestinal peptide (VIP) or Nα-syn both in CFA/iFA	MPTP T cell transfer from VIP or Na-syn immunized animals or Treg	Lymphocytes	*N*-α-syn induce Th17 inflammatory T cells, Th2 rescued neuron cell death and inhibit Treg functionVIP T cells decreased Mac1 expression and cell deathVIP induced Foxp3 and IL-10 expressionTreg rescued neurons	Reynolds et al., 2010
α-syn in CFA/iFA CFA/iFA alone	AAV-αsyn SN expressing rats previously immunized with α-syn (150 μg in CFA, 10 wks previous 100 μg in iFA, 6 wks previous)	Peripheral immune system	Induction of GDNF in strAccumulation of Treg in SN + strReduction of pathological α-syn aggregatesDistinct microglia activation (MHC II+, CD4+)α-syn specific IgG deposition on neurons	Sanchez-Guajardo et al., 2013
α-syn in CFA/iFA CFA/iFA alone	α-syn overexpressing mice under the PDGF-β promoter 8 week vaccination scheme (80 μg in CFA, two weeks after 80 μg in iFA, then once a month)	Peripheral immune system	Increased synaptophysin levelsAntibody deposition on neurons (intercellular)Antibody-mediated lysosomal degradation of α-syn	Masliah et al., 2005
Passive immunization with α-syn antibody	α-syn overexpressing mice under the PDGF-β promoter	Neurons	Reduced astroglia activationClearance of pathological α-syn aggregates	Masliah et al., 2011

Strategies aimed at harnessing the inflammatory response in pre-clinical models of PD have included use of anti-inflammatory gene therapy approaches: nigral overexpression of a dominant negative TNF molecule to block native TNF signalling has been shown to effectively protect neurons from 6-OHDA-induced cell death even after delayed administration (McCoy et al., 2008; Harms et al., 2011) and GDNF delivery has been shown to have a protective effect in pre-clinical animal models of nigral cell death (reviewed in Lindvall and Wahlberg, 2008), although it did not prove effective in rAAV-α-syn overexpression models (Lo Bianco et al., 2004; Decressac et al., 2011). Another approach has been the use of NSAIDs (non-steroid anti-inflammatory drugs) as their use seems to reduce the risk of PD development (Chen et al., 2005; Etminan et al., 2008; Samii et al., 2009). Anti-inflammatory compounds, such as naloxane, minocycline and dexamethasone can reduce microglia activation and neuronal damage in different models of nigral degeneration (Liu et al., 2000; Lu et al., 2000; Tikka et al., 2001; Castano et al., 2002; Wu et al., 2002). In particular, minocycline, a broad-spectrum tetracycline derivative, inhibits microglia and T-cell activation thereby attenuating inflammatory factor production (Giuliani et al., 2005); yet its ability to block cell MPTP-induced nigral degeneration is variable and may depend on dose and frequency of administration (Du et al., 2001; Wu et al., 2002; Yang et al., 2003; O’Callaghan et al., 2008). Minocycline has also been shown to attenuate nigral degeneration induced by 6-OHDA (He et al., 2001; Quintero et al., 2006; Koprich et al., 2008), likely through inhibition of H_2_O_2_ radicals (Lin et al., 2003). The timing of administration appears to be important, as DA cell loss was not as evident when minocycline was given after 6-OHDA injections (Quintero et al., 2006). Alternatively, the potential neuroprotective compounds may be hydrogen sulphide-releasing l-DOPA derivatives that reach the brain and reduce the level of IL-6/TNF and NO from microglia (Lee et al., 2010b). More specific blockade of inflammation has been achieved successfully with inhibitors of COX-2 (Sanchez-Pernaute et al., 2004), which has been shown to be increased in PD SN (Teismann et al., 2003). COX-2 can induce DA oxidation and NFκB-induced inflammatory responses in microglia (Schwieler et al., 2006; Hsieh et al., 2011); additionally it induces prostaglandin production that triggers the peripheral immune system (Font-Nieves et al., 2012).

Strategies aimed at modulating the peripheral immune system have been mainly designed to prime T-cells *in vivo* with different agents and then transfer them to the periphery of animal models that have undergone a procedure to induce nigral cell death. These agents have been both specific for DA neurons/pathology (TH, α-syn) or known to induce a protective T-cell phenotype VIP (vasoactive intestinal peptide), GA (galatiramer acetate), BCG (Bacille Calmette–Guérin), myelin oligodendrocyte glycoprotein (Benner et al., 2004; Kurkowska-Jastrzebska et al., 2005; Armentero et al., 2006; Laurie et al., 2007; Reynolds et al., 2007, 2010; Yong et al., 2011 (Sanchez-Guajardo et al., 2013). Most interestingly, CFA (complete Freund's adjuvant), a commonly used immunopotentiator by itself seems to induce a protective immune response and naturally all vaccination strategies have included it (Armentero et al., 2006). Support for the effect of CFA in immunotolerance induction comes from an experiment in which live BCG, which is related to *Mycobacterium tuberculosis* found in CFA, induced neuroprotective effects (Yong et al., 2011). As T-cells were able to prevent DA cell death and modify microglia activation independently of the immunogen used, these immunization strategies suggest that they have achieved tolerance towards processes happening in the PD models more than they point to a specific T-cell effector response. Indeed we have observed that α-syn immunization prior to rAAV–α-syn overexpression in the nigrostriatal system modified microglia profiles in SN (Sanchez-Guajardo et al., 2013). Another common factor in all these strategies is that they induce T_reg_ (regulatory T-cells)/tolerance: GA is a TCR agonist that blocks MHC II function and induces T_reg_ cells, VIP is also known for inducing T_reg_, α-syn recruited T_reg_ cells to the nigrostriatal system. Indeed, T_reg_ transfer into MPTP-treated animals attenuated loss of nigral DA neurons (Reynolds et al., 2010). It has also been shown that nitrated α-syn inhibits T_reg_ suppressive function, which implies that during PD the immune response to α-syn may not be tightly regulated by T_reg_ and a chronic activation of the immune system can take place.

Transgenic mice overexpressing α-syn that have been immunized with α-syn or with antibody specific for α-syn also demonstrated amelioration of pathology but no effect on microglia (Masliah et al., 2005, 2011). This could be due to the fact that T_reg_ cells specific for pathological α-syn and tolerance against it were already induced during T-cell thymic development, so T-cells are already unreactive; however, the limitation of those studies is that no characterization of the T-cell compartment was performed. Nevertheless, these studies did demonstrate that humoral immunity is crucial for α-syn clearance, which implies B cell activation and Th cell involvement if memory B cells are to be induced. In summary, modulation of the peripheral immune system to induce T-cells capable of inducing tolerance to α-syn-mediated neurodegeneration and modulation of the microglia response during PD is a promising and potentially potent therapeutic strategy that could be ready for the clinic in the next 5 years. One great advantage of this kind of immunomodulation approach would be the ability to circumvent the need to act directly on the brain itself and could also potentiate the beneficial aspects of the microglia response that may be occurring in PD instead of inhibiting inflammatory responses altogether.

## CONCLUSIONS

**Figure 1:**
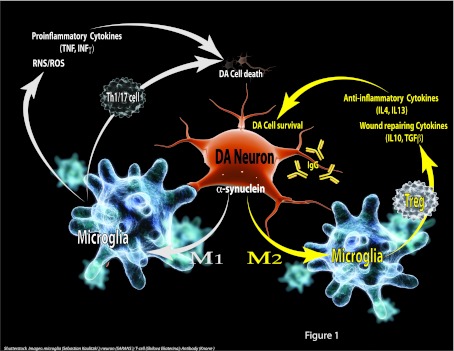
The specific interaction of microglia with extraneuronal α-syn influences the balance between M1 compared with M2 activation states and determines the extent of DA neuron survival compared with neuron death α-Syn from DA neurons is taken up by microglia. Initially, these M2 microglia produce anti-inflammatory and tissue repair factors to promote survival, as well as modify the BBB by producing IL-6 and IL-1, which allows for infiltration of peripheral immune cells. Over time, the accumulation of α-syn or the recognition of α-syn by microglia through different mechanisms (synapse snatching, phagocytosis, pinocytosis, TLR2&4, Mac1 and FcγR), leads to a phenotypic shift in their response from M2 to M1. M1 microglia produce pro-inflammatory cytokines and RNS/ROS that are toxic to DA neurons, and recruit peripheral immune cells with a Th1 and/or Th17 phenotype. This toxic environment created by microglia is a perfect storm that could facilitate the progressive loss of DA neurons observed in PD. Therapeutic strategies should consider ways to aid microglial processing of α-syn in a way that favours an M2 microglial phenotype, as this would favour the induction of T_reg_ and α-syn-specific IgG production, both of which have been shown to be protective and help to clear α-syn deposition.

In conclusion, we have reviewed here the overwhelming evidence that supports a role for neuroinflammation in PD and PD-like neurodegeneration. The number of activated microglia in post-mortem PD brains, cytokine levels in CNS and blood, the presence of IgG and the infiltration of T-cell in CNS suggest that this process involves not only the local immune system, but also the peripheral immune system. The reports of neuroprotection based on different strategies targeting various inflammatory mechanism or pathways at the central or peripheral level suggest that such therapeutic approaches may prove beneficial in PD patients to delay or attenuate onset of disabling motor symptoms. However, we should keep in mind that these strategies should not only aim for halting neuroinflammation or microglia activation, but should instead focus on modulating the response of these cells by boosting an M2 compared with an M1 phenotype or a Th2/T_reg_ compared with Th1 phenotype (Figure 1). Within this context, we have highlighted the importance of α-syn as an initiator of pathogenesis but also as a factor that contributes to the persistent microglia activation. Therefore neuroprotective strategies should not only aim to control the deleterious effects of microglia activation, but must also address the neuronal processing of α-syn and the clearance of the extracellular α-syn, which is most efficiently done by microglia. More than likely, a successful outcome will require a combination of immunomodulatory strategies rather than one targeted solely at single inflammatory factors elevated in PD and implicated in disease pathophysiology.

## FINANCIAL DISCLOSURES

M. Romero-Ramos and V. Sanchez-Guajardo have no financial stake or holdings to declare. M.G. Tansey was an ex-employee of Xencor Inc., a biotherapeutics company. She has no significant financial stake in the company and is not a consultant. C. J. Barnum has no financial stake in any company or holdings to declare.
